# Responses of Soil Microbial Communities in an Alkalized Meadow Soil to Biochar Addition

**DOI:** 10.3390/microorganisms13061228

**Published:** 2025-05-27

**Authors:** Tingting Gao, Ying Zhang, Zhenbo Cui, Chengyou Cao

**Affiliations:** 1 College of Life and Health Sciences, Northeastern University, Shenyang 110169, China; gaotingting@mail.neu.edu.cn (T.G.); zhangying@mail.neu.edu.cn (Y.Z.); cuizhenbo@mail.neu.edu.cn (Z.C.); 2 Liaoning Province Key Laboratory of Bioresource Research and Development, Northeastern University, Shenyang 110169, China

**Keywords:** soil bacterial community, soil fungal community, functional gene, biochar, grassland improvement

## Abstract

Biochar is increasingly being applied to improve various degraded soils. However, studies on its use in ameliorating saline–alkaline grasslands remain limited. This study conducted experimental trials using soil collected from an alkalized meadow grassland in the Horqin Steppe, applying biochar with the application rates of 0, 1.5, 3.0, and 4.5 kg/m^2^ in planting boxes. The objectives were to evaluate the effects of biochar addition on soil properties and microbial community and to explore the feasibility of using biochar for alkalized grassland improvement. Biochar addition to alkalized meadow soil enhanced the biomass of planted *Astragalus adsurgens* and improved soil properties. Soil bulk density was reduced; porosity, moisture content, and field moisture capacity significantly increased; soil nutrients were significantly ameliorated. Simultaneously, soil enzyme activities, including urease, phosphomonoesterase, protease, and polyphenol oxidase, significantly increased. Biochar application altered the microbial community structures in the alkalized meadow soil, primarily through the shifts in the relative abundance of dominant taxa rather than the fundamental changes in dominant phyla or genera. Biochar addition significantly raised the abundance of *phoD*- and *nifH*-harboring microorganisms, suggesting the enhancement in functions of soil N fixation and P transformation. Key factors influencing bacterial community structure included electrical conductivity, total P, total K, bulk density, and available K, whereas fungal communities were primarily affected by bulk density, porosity, and available N. Excessive biochar application can diminish its yield-enhancing effects, and the recommended biochar application rate for alkalized meadow grasslands in practice is 1.5 kg/m^2^. These findings are expected to provide experimental evidence for utilizing biochar in degraded grasslands improvement.

## 1. Introduction

Biochar is a carbon-rich, highly stable solid material produced through the thermochemical conversion of organic biomass under oxygen-limited conditions. With its abundant micropores and a large specific surface area [1], biochar serves as an effective soil amendment in agricultural production to enhance crop yields [2]. Studies demonstrate that biochar application effectively improves both acidic and alkaline degraded soils [3,4]. In recent years, research on biochar’s role in ameliorating saline–alkali soils has increased significantly. Biochar demonstrates dual remediation mechanisms in saline–alkali soil ecosystems through physicochemical interactions and nutrient supplementation. Primarily, it induces the physical dilution of salt ions while facilitating cation exchange processes, effectively modifying soil structure and ionic composition [5,6]. Simultaneously, as a multifunctional amendment, biochar serves as a slow-release reservoir of essential macronutrients, including nitrogen (N), phosphorus (P), potassium (K), and organic carbon (C), thereby enhancing soil fertility [7]. These synergistic modifications collectively improve the edaphic environment by optimizing soil porosity, adjusting pH levels, and increasing cation exchange capacity. The resultant amelioration of physicochemical parameters and nutrient bioavailability establishes favorable microhabitats for soil microbiota. Such environmental optimization subsequently modulates microbial community dynamics through stimulating metabolic enzyme activity, restructuring phylogenetic diversity, and altering functional gene expression patterns [5,7]. Previous studies indicate that biochar addition increases the relative abundance of N-fixing microorganisms [8,9], denitrifying bacteria [10], and phosphate-solubilizing bacteria [11] in salinized soils, and suggests that biochar application significantly alters the composition pattern of microbial phylum [5,12] and drives soil microbial communities toward salt–alkali tolerance, drought resistance, and oligotrophic adaptation [12,13]. However, some studies report that biochar amendment does not affect soil microbial diversity [14]. The responses of the diversities and structures of microbial communities to biochar addition in saline–alkali soils vary significantly across studies, with no consistent conclusions reached. Current research remains inconclusive regarding biochar’s impacts on soil microbial communities, primarily due to two factors. First, biochar’s intrinsic complexity manifests through dual mechanisms: its porous structure and high specific surface area can directly modify soil physicochemical properties and provide microbial refuge against predation [15], and toxic compounds (polycyclic aromatic hydrocarbons, furans, and phenols) generated during production may inhibit microbial growth [16]. Second, experimental variability arises from the differences in application duration, biochar dosage, and site-specific environmental conditions across studies.

Located in temperate grassland zone, the Horqin Sandy Lands of China historically featured a forest–steppe landscape with abundant lakes and luxuriant vegetation, serving as a traditional pastoral region. This area predominantly supports meadow grasslands that function as primary hay-producing grasslands due to their relatively fertile soils, favorable moisture conditions, rich plant diversity, and high biomass productivity. However, rapid population growth and livestock expansion have intensified grassland utilization, and has been superimposed by a climate warming-drying trend under global change. These pressures have triggered the severe salinization–alkalization of grassland, resulting in a dramatic decline in forage yield and significant shifts in plant community composition. Vegetation degradation and productivity loss are closely linked to soil salinization–alkalization and associated the alterations in soil physicochemical properties and biological activity. Consequently, effective grassland restoration in this region requires fundamental soil improvement. Conventional rehabilitation methods involving the soil spring plowing and harrowing followed by reseeding with leguminous or graminaceous forage species have demonstrated moderate success. Given biochar’s proven soil amendment properties, its application to degraded and alkalized meadow soils may offer enhanced remediation potential.

This study collected typical alkalized meadow soil from the Horqin grassland and assessed the improvement effects of biochar application through controlled experiments. The aims of present research are: (1) to examine the impacts of biochar addition on edaphic physicochemical attributes and biological activity; (2) to characterize the responses of soil microbial communities and key functional microbial populations (including ammonia-oxidizing, N-fixing, and organic P-mineralizing bacteria) to biochar amendment; and (3) to analyze the relationships between microbial community structure and soil factors under biochar application. The results are expected to provide an in-depth understanding of the restoration mechanisms underlying biochar application in degraded grasslands, while providing theoretical foundations for implementing grassland rehabilitation practices in this region.

## 2. Materials and Methods

### 2.1. Study Location Description

The study area is situated in Wulanaodu Village (43°02′ N, 109°39′ E), Wengniute county, Inner Mongolia Autonomous Region, located in the western sector of the Horqin Sandy Land in northeastern China. This region experiences a temperate continental monsoon climate characterized by distinct seasonal variations. Dominant meteorological characteristics include an annual mean temperature of 6.2 °C, 130 frost-free days, and pronounced diurnal thermal fluctuations. The hydrological regime demonstrates significant interannual variability, with mean annual precipitation measuring 340.5 mm concentrated predominantly (70–80%) during the May–September period when the rainy season coincides with the warmest thermal conditions. This precipitation pattern contrasts sharply with the considerable annual potential evaporation of 2500 mm. Aeolian activity is particularly pronounced in winter and spring seasons, sustained by persistent winds averaging 4.5 m/s with frequent dust events. The landscape exhibits a dynamic succession of aeolian landforms comprising mobile dunes, semi-fixed dunes, and stabilized dunes, interspersed with interdune lowland ecosystems. The Wulanaodu region supports extensive natural meadow grasslands with high plant diversity. Typical herbaceous species include *Aneurolepidium chinense*, *Spodiopogon sibiricus*, *Arundinella hirta*, *Potentilla chinensis*, *Chloris virgate*, *Phragmites communis*, and *Suareda glacuca*. Since the 1970s, climate change coupled with habitat aridification and overgrazing has led to progressive degradation, salinization, and significant productivity decline in these grasslands. Improving alkalized meadow soils has been identified as an effective approach to enhance grassland productivity in this area.

### 2.2. Experimental Design and Soil Sampling

The biochar amendment experiment was conducted at the Wulanaodu Desertification Experimental Station affiliated with the Institute of Applied Ecology, Chinese Academy of Sciences. Surface soil samples (0–20 cm depth) were collected from adjacent alkalized meadow grasslands. Commercially available corn stalk-derived biochar (Liaoning Golden Future Agriculture Technology Co., Ltd., Benxi, China), produced via high-temperature anaerobic pyrolysis (500 °C), was homogenized with soil matrices. Four biochar application rates were tested: 0 (control), 1.5, 3.0, and 4.5 kg/m^2^ (designated as AM0, AM1.5, AM3.0, and AM4.5 respectively), using cultivation boxes (40 × 40 × 30 cm) with triplicate randomized blocks. Corresponding biochar quantities of 0, 0.72, 1.44, and 2.16 kg per box (equivalent to 0, 1.5, 3.0, and 4.5 kg/m^2^, respectively) were thoroughly mixed into the soils. The biochar–soil mixtures were hydrated to field capacity and stabilized for 30 days under controlled conditions. Perennial legume *Astragalus adsurgens* was sown with final density maintained at two seedlings per box, accompanied by standardized agronomic practices. The experiment lasted two years. Aboveground biomass was quantified at phenological maturity (mid-September) over two consecutive growing seasons. Second-year soil samples were divided into three parts: air-dried for physicochemical analysis, stored at 4 °C for enzyme activity determination, and preserved at −80 °C for DNA extraction and high-throughput sequencing. The characterized biochar exhibited a pH of 7.98 with specific physical properties: a surface area of 2.89 m^2^/g (BET measurement) and mesoporous structure evidenced by 188.5 nm average pore diameter. Its elemental composition included C (70.38%), N (1.53%), P (0.78%), K (1.68%), Mg (1.25%), Ca (0.58%), S (0.65%), and Fe (0.11%).

### 2.3. Soil Property Determinations

Soil pH and electrical conductivity (EC) were determined using 1:2.5 and 1:5 soil-water suspensions, respectively. Soil hydraulic parameters, including field water holding capacity (FC), bulk density (BD), and porosity (SP), were measured through the cutting ring method (5 cm diameter × 5 cm height). Soil organic matter (SOM) content was quantified via the potassium dichromate–sulfuric acid (K_2_Cr_2_O_7_-H_2_SO_4_) oxidation method. Total N (TN) analysis was performed using semi-micro Kjeldahl digestion coupled with an automatic N analyzer (K9860, Haineng Scientific Co., Ltd., Jinan, China). Soil available P (AP) and total P (TP) were determined by the Olsen and Dean method. Potassium (K) analysis included available K (AK) and total K (TK) determinations using atomic absorption spectroscopy. Ammonium N (NH_4_⁺-N) quantification was conducted via salicylic acid colorimetry at 460 nm. Automated analyses for TP, AP, and NH_4_⁺-N were performed using a discrete analyzer (CleverChem 380, Dechentreiter GmbH, Hamburg, Germany). All analytical procedures followed the standard protocols described in ISSCAS [17].

Enzyme activity determinations were conducted as follows: β-glucosidase: Modified Xu-Zheng method [18]; Dehydrogenase: ISSCAS standard protocol [19]; Urease: Substrate-specific method with urea hydrolysis quantification at 460 nm [20]; Protease: Ladd–Butler procedure [21]; Phosphomonoesterase: Schinner’s colorimetric approach [22]; and Polyphenol oxidase: Perucci’s spectrophotometric method [23].

### 2.4. Soil DNA Extraction, 16S rRNA/ITS Gene Sequencing, and Functional Gene Quantification

Genomic DNA was extracted from soil samples of the AM0, AM1.5, AM3.0, and AM4.5 treatments using a Soil DNA Quick Extraction Kit (Bioteke, Wuxi, China). The V3-V4 hypervariable regions of bacterial 16S rRNA and fungal *ITS* genes were amplified and sequenced on an Illumina MiSeq platform (Shanghai Personal Biotechnology Co., Ltd., Shanghai, China). Raw sequences underwent quality filtering and chimera removal. High-quality sequences were clustered into operational taxonomic units (OTUs) at 97% sequence similarity. Taxonomic classification was performed by BLAST alignment against the NCBI GenBank database (http://blast.ncbi.nlm.nih.gov/Blast.cgi, accessed on 25 October 2022). All sequencing data were deposited in the NCBI Sequence Read Archive (Accession: PRJNA706575). Functional gene abundances were quantified via qPCR: Ammonia-oxidizing bacteria: *amoA* gene (Primers: F-GGGGTTTCTACTGGTGGT/R-ATCATGGT(C/G)CTGCCGCG) [24]; N-fixing bacteria: *nifH* gene (Primers: F-TGCGAYCCSAARGCGAC/R-ATSGCCATCATYTCRCCGGA) [25]; and Organic phosphorus-mineralizing bacteria: *phoD* gene (Primers: F-TGGGAYGATCAYGARGT/R-CTGSGCSAKSACRTTCCA) [26]. Amplifications were conducted using a Q5 Real-Time PCR System (Applied Biosystems, Waltham, MA, USA) with SYBR Green detection. Standard curves were generated from plasmid dilutions (10^3^–10^9^ copies/μL) for absolute quantification.

### 2.5. Statistical Analysis

Treatment effects on aboveground biomass, soil properties, and functional gene abundances were analyzed by a one-way ANOVA with LSD post hoc tests (SPSS 18.0). Pearson correlation analysis elucidated relationships between gene abundances and soil parameters. Simultaneously, the responses of the above parameters to the amount of biochar addition were fitted by the linear regression model (SPSS 18.0). *p* < 0.05 was considered statistically significant. An unweighted pairwise cluster analysis was used to compare the differences in the structures of soil microbial communities of the treatments. LDA effect size (LEfSe) was used to detect the differentiated microbial taxa among different treatments. A canonical correspondence analysis (CCA) by CANOCO 5.0 (Biometris-Plant Research International, Wageningen, The Netherlands) was performed to determine the main soil factors significantly affecting microbial communities.

## 3. Results

### 3.1. Effects of Biochar Application on Biomass and Soil Properties

The two-year experimental results show that the addition of an appropriate amount of biochar to alkalized soil significantly enhanced the aboveground biomass of cultivated *Astragalus adsurgens*. In the first year, the biomass values of *A. adsurgens* under the AM1.5, AM3.0, and AM4.5 treatments were 1.23, 1.55, and 1.42 times that of the control (AM0), respectively. The biomass exhibited a statistically significant linear regression association with the application rate of biochar (*p* < 0.001). During the second experimental year, biomass production exhibited a dose-dependent response to AM treatments. The AM1.5 and AM3.0 treatments significantly enhanced biomass accumulation, reaching 2.59-fold and 1.62-fold of the AM0 baseline, respectively. Notably, this positive trend was reversed under the AM4.5 treatment, which showed a 20.6% reduction in biomass compared to the control group (Table 1). This nonlinear response pattern suggests the existence of an optimal threshold for AM treatment effect.

The physicochemical properties of soils under different treatments in the second experimental year are presented in Table 2. As shown in Table 2, biochar addition significantly influenced the physicochemical properties and nutrient levels of the alkalized soil. Soil moisture (SM), SP, FC, EC, SOM, TN, and NH_4_-N, AP, and AK all exhibited significant linear increasing trends, while BD showed a significant linear decreasing trend. Changes in soil pH, TP, and TK did not reach significant levels. Compared to AM0, the addition of varying biochar dosages increased physical property indicators (SM, SP, FC, and EC) by 1.42–1.76, 1.08–1.18, 1.16–1.33, and 1.32–1.42 times, respectively. Soil nutrients, including OM, TN, AP, and AK, were enhanced to 1.49–1.94, 1.45–1.69, 1.10–1.23, 1.70–2.72, and 1.39–1.46 times those of the control, respectively. In contrast, soil BD decreased from 1.60 g/cm^3^ to 1.52, 1.44, and 1.42 g/cm^3^. These results indicate that biochar addition effectively improves soil physical properties and enhances soil nutrients, and the improvement effects increase with the application rate.

### 3.2. Impact of Biochar Addition on Soil Microbial Activity

The activities of six enzymes (dehydrogenase, protease, urease, polyphenol oxidase, phosphomonoesterase, and glucosidase) were measured, and the results are listed in Table 3. Biochar amendment to alkalized meadow soil exerted differential effects on various soil enzymatic activities. Notably, biochar application significantly stimulated protease, urease, polyphenol oxidase, and phosphomonoesterase activities, while conversely suppressing dehydrogenase and β-glucosidase activities compared to the controls. The AM1.5 treatment showed the highest activities of protease, urease, and phosphomonoesterase, which were 1.43, 2.96, and 1.56 times that of the control (AM0), respectively. The activities of the three enzymes in both AM3.0 and AM4.5 treatment groups exhibited significantly elevated levels (*p* < 0.05) compared to the control group. The maximum polyphenol oxidase activity was observed in the AM3.0 treatment (Table 3). Significant linear regression relationships were identified between biochar application rate and the activities of dehydrogenase, glucosidase, and polyphenol oxidase.

### 3.3. Effects of Biochar Amendment on the Quantities of Functional Microorganisms Involved in N Fixation, Ammonia Oxidation, and P Transformation

**Figure 1:**
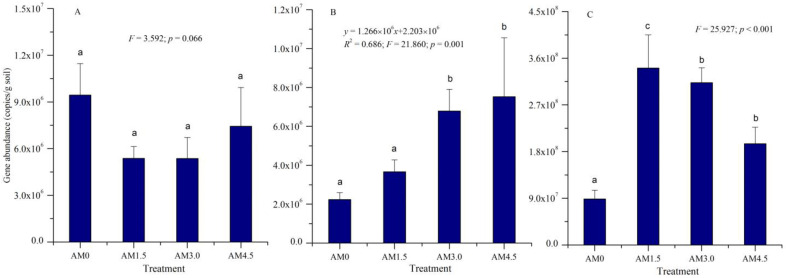
The abundance of functional genes of microbial community. AM0: Control; AM1.5, AM3.0, and AM4.5: 1.5, 3.0, and 4.5 kg/m^2^ treatments, respectively. The regression analysis results for (**B**) are presented, including the *R*^2^ value, *F*-statistic, and corresponding *p*-values. Columns labeled with different letters indicate statistically significant differences at α = 0.05 level. (**A**): *amoA* gene; (**B**): *nifH* gene; (**C**): *phoD* gene.

The copies of *amoA*, *nifH*, and *phoD* genes in soil samples under different treatments were quantified via absolute Q-PCR to estimate the abundances of N-fixing bacteria, ammonia-oxidizing bacteria, and organic P-mineralizing bacteria, respectively. The *nifH* gene copy numbers were quantified within the range of 2.24–7.52 × 10^6^ per gram of desiccated soil across experimental conditions (Figure 1). The AM3.0 and AM4.5 treatments exhibited significantly higher *nifH* gene abundances compared to AM0 and AM1.5 (*p* < 0.05). A significantly positively linear relationship between the abundance of the *nifH* gene and biochar application rate was observed (*p* = 0.001).

Similarly, the *phoD* gene abundance exhibited significant variations across experimental treatments (*p* < 0.001), ranging from 8.85 × 10^7^ to 3.41 × 10^8^ copies per gram of dry soil. Notably, biochar-amended treatments demonstrated statistically significant increases in *phoD* gene copies compared to the control group. The highest *phoD* gene abundance was observed in the AM1.5 treatment. In contrast, no significant differences were detected in *amoA* gene abundance across treatments, which ranged from 5.36 × 10^6^ to 9.43 × 10^6^ copies/g dry soil. The *nifH* gene abundance exhibited significant positive correlations with SM, BD, porosity, TN, and polyphenol oxidase activity (*p* < 0.05; Pearson correlation coefficient: 0.641–0.795). The *phoD* gene abundance showed significant positive correlations with soil EC, TN, OM, AK, urease activity, and polyphenol oxidase activity (Pearson correlation coefficient: 0.586–0.842).

### 3.4. Response of Soil Bacterial Community to Biochar Addition

Illumina MiSeq high-throughput sequencing yielded a total of 755,214 high-quality 16S rRNA gene sequences. Clustering at 97% similarity identified 9713 operational taxonomic units (OTUs) in the control (AM0), while the biochar-amended treatments (AM1.5, AM3.0, and AM4.5) showed higher OTU numbers of 10,916, 10,598, and 9944, respectively, indicating enhanced bacterial diversity with biochar addition. The taxonomic analysis revealed a total of 31 bacterial phyla, 100 classes, 231 orders, 382 families, and 566 genera through sequence classification. Dominant phyla included Proteobacteria, Actinobacteria, Acidobacteria, Cyanobacteria, Bacteroidetes, Chloroflexi, Patescibacteria, Firmicutes, Verrucomicrobia, Gemmatimonadetes, Rokubacteria, and Planctomycetes. Proteobacteria and Actinobacteria were absolutely dominant across all samples, with mean relative abundances of 30.78% and 26.10%, respectively. Although variations in the relative abundances of dominant phyla were observed among treatments, none reached statistical significance.

**Figure 2:**
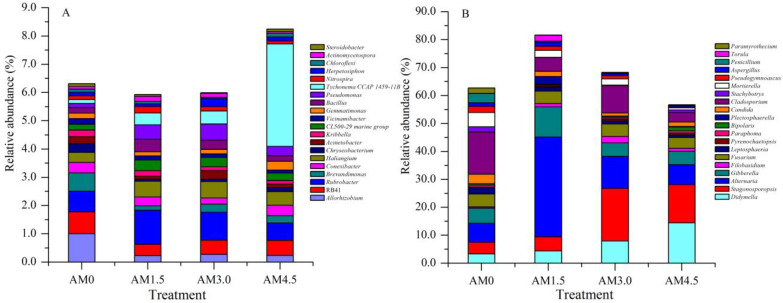
The relative abundance of soil dominant microbial genera (top 20). AM0: Control; AM1.5, AM3.0, and AM4.5: 1.5, 3.0, and 4.5 kg/m^2^ treatments, respectively. (**A**): Soil bacterial community; (**B**): soil fungal community.

**Figure 3:**
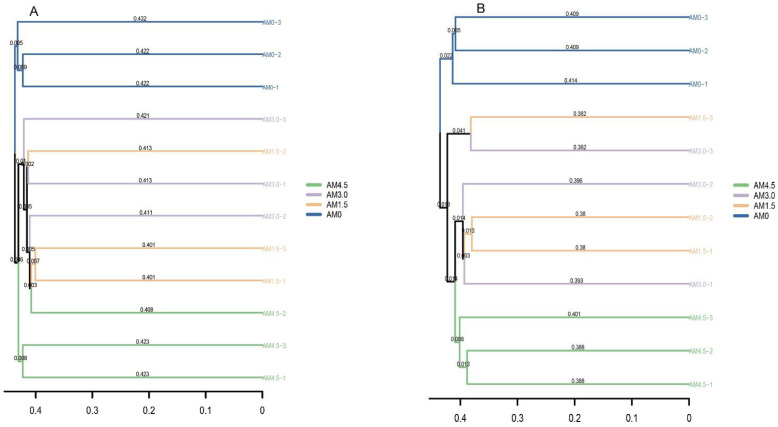
Cluster analysis of soil bacterial (**A**)/fungal (**B**) communities according to different treatments. AM0: Control; AM1.5, AM3.0, and AM4.5: 1.5, 3.0, and 4.5 kg/m^2^ treatments, respectively.

The basic composition of dominant bacterial genera in alkalized meadow soils treated with varying biochar application rates was similar, but their relative abundances differed significantly across treatments. In the biochar-amended treatments, the relative abundances of genera such as RB41, *Chryseobacterium*, and *Vicinamibacter* were significantly lower than those in the control (AM0), while *Haliangium* and *Luteitalea* showed significantly higher relative abundances. In the AM1.5 treatment, genera including *Rubrobacter*, *Bacillus*, and *Actinomycetospora* exhibited significantly higher relative abundances compared to other treatments, whereas *Nannocystis* and *Patulibacter* had significantly lower relative abundances. In the AM3.0 treatment, *Thermus* and *Luteolibacter* were more abundant than in other treatments, while *Conexibacter* and *Gemmatirosa* were significantly reduced. In the AM4.5 treatment, the relative abundance of *Tychonema* CCAP 1459-11B increased markedly, whereas *Chthoniobacter* and *Nocardioides* decreased significantly (Figure 2A). These results demonstrate that biochar addition significantly altered bacterial community structure, characterized by dynamic shifts in the dominance of key genera. The UPGMA clustering analysis grouped all samples into three clusters: AM0 and AM4.5 samples formed distinct individual clusters, while AM1.5 and AM3.0 samples clustered together (Figure 3A). This pattern highlights both the pronounced impact of biochar on the taxonomic profiles of the bacterial community and the similarity between AM1.5 and AM3.0 treatments.

**Figure 4:**
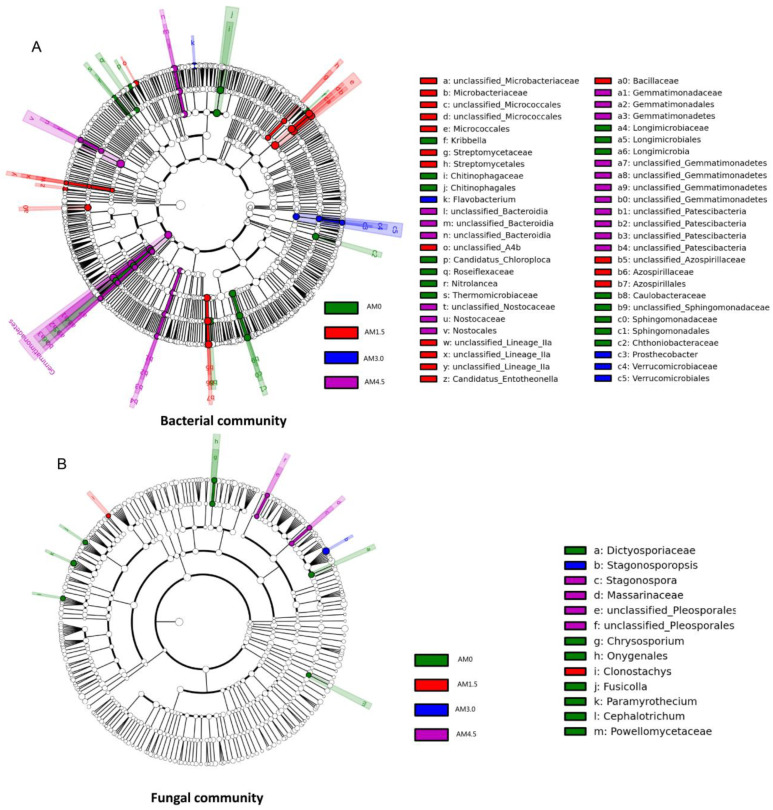
LEfSe analysis of soil bacterial (**A**)/fungal (**B**) community of different treatments. AM0: Control; AM1.5, AM3.0, and AM4.5: 1.5, 3.0, and 4.5 kg/m^2^ treatment, respectively.

As illustrated in Figure 4A, a LEfSe (Linear Discriminant Analysis Effect Size) analysis was performed to identify and characterize phylogenetically distinct microbial taxa exhibiting significant differential abundance across experimental treatments. Significantly differential taxa were identified in all treatments: AM0: Chitinophagales (Chitinophagaceae), Sphingomonadales (Sphingomonadaceae), Caulobacteraceae, Chthoniobacteraceae, Longimicrobiales (Longimicrobiaceae), Thermomicrobiaceae, Roseiflexaceae, *Kribbella*, *Nitrolancea*, and *Candidatus Chloroploca*; AM1.5: Micrococcales (Microbacteriaceae), Azospirillales (Azospirillaceae), Bacillaceae, Anaerolineae (A4b), Streptomycetales (Streptomycetaceae), *Lineage*, and *Candidatus Entotheonella*; AM3.0: Verrucomicrobiales (Verrucomicrobiaceae and *Prosthecobacter*) and *Flavobacterium*; and AM4.5: Bacteroidia, Gemmatimonadales (Gemmatimonadaceae), Nostocales (Nostocaceae), and Patescibacteria (Figure 4A). The LEfSe results further confirm that biochar addition significantly influenced the bacterial community structure by altering the dominance of specific taxa.

### 3.5. Response of Soil Fungal Community to Biochar Addition

A total of 510,996 *ITS* gene sequences were obtained from all samples. Clustering at 97% similarity identified 509 OTUs in the control (AM0), while AM1.5, AM3.0, and AM4.5 showed higher OTU numbers of 750, 662, and 604, respectively, indicating enhanced fungal diversity with biochar addition. The sequencing data were taxonomically assigned to six hierarchical levels, spanning 12 phyla, 32 classes, 76 orders, 163 families, and ultimately 293 genera. The dominant fungal phyla across treatments were Ascomycota, Basidiomycota, Blastocladiomycota, Chytridiomycota, Mortierellomycota, and Mucoromycota, though their relative abundances varied significantly. Ascomycota was overwhelmingly dominant in all treatments, with mean relative abundances of 72.9%, 88.3%, 74.7%, and 71.8% in AM0, AM1.5, AM3.0, and AM4.5, respectively. This was followed by Basidiomycota (3.55–10.6%) and Mortierellomycota (0.81–5.14%). Biochar addition significantly altered the relative abundances of dominant fungal genera. It notably reduced the abundances of *Stachybotrys*, *Penicillium*, and *Chrysosporium*, while increasing those of *Filobasidium* and *Pyrenochaetopsis*. Dominant genera also exhibited dynamic shifts across treatments. For example, in AM1.5, *Acremonium*, *Gibberella*, and *Plectosphaerella* showed higher relative abundances compared to AM3.0 and AM4.5, whereas *Coniothyrium* and *Exserohilum* were less abundant (Figure 2B). The UPGMA clustering analysis of fungal communities yielded results similar to those of bacterial communities, with AM0 and AM4.5 forming distinct clusters, while AM1.5 and AM3.0 were clustered together (Figure 3B). LEfSe identified significantly divergent fungal taxa across treatments: AM0: Onygenales, Dictyosporiaceae, Powellomycetaceae, *Paramyrothecium*, *Chrysosporium*, *Fusicolla*, and *Cephalotrichum*; AM1.5: *Clonostachys*; AM3.0: *Stagonosporopsis*; and AM4.5: Pleosporales, Massarinaceae, and *Stagonospor* (Figure 4B).

### 3.6. Relationships Between Microbial Community Shifts and Edaphic Properties

**Figure 5:**
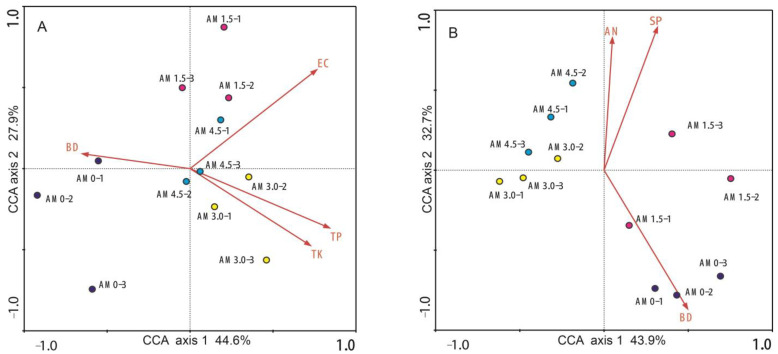
CCA between microbial community structure and soil factors. (**A**): Soil bacterial community; (**B**): soil fungal community. The red arrows denote soil properties, with experimental treatments distinguished by distinct color coding. EC: electrical conductivity; BD: bulk density; SP: soil porosity; TP: total P; TK: AN: NH_4_^+^-N. AM0: Control; AM1.5, AM3.0, and AM4.5: 1.5, 3.0, and 4.5 kg/m^2^ treatments, respectively.

CCA was employed to elucidate the multivariate relationships between microbial community assemblages (bacteria and fungi) and environmental factors (including SM, SP, FC, EC, OM, TN, NH_4_-N, AP, and AK). The CCA ordination plot revealed distinct differences in bacterial and fungal community structures across treatments. For bacterial communities, 72.5% of total variance were explained (axis 1: 44.6%; axis 2: 27.9%), while for fungal communities, the value was 76.6% (axis 1: 43.9%; axis 2: 32.7%) (Figure 5). Monte Carlo permutation tests identified soil factors significantly influencing bacterial community structure: EC, TP, TK, BD, and AK, with variation explanation rates of 18.0%, 17.0%, 15.0%, 14.0%, and 14.0%, respectively. For fungal communities, key factors were BD, SP, and NH_4_-N, explaining 19.0%, 18.0%, and 16.0% of the variation, respectively. In the CCA ordination, triplicate samples from the same treatment clustered closely, whereas samples from different treatments formed separate clusters. The distribution of treatments across distinct quadrants in the CCA biplot further highlighted the differences in bacterial and fungal community structures.

## 4. Discussion

### 4.1. Effects of Biochar Amendment on Forage Biomass in Alkalized Meadow Soil

Considerable experimental research has verified that biochar significantly enhances plant biomass by improving soil physicochemical properties [2,3]. However, some studies have reported minimal or even negative effects [27,28]. Overall, the impacts of biochar on plant growth vary depending on its properties, application duration, soil type, and crop species [6]. In this study, the overground biomass of planted *A. adsurgens* in the AM1.5 and AM3.0 treatment groups significantly exceeded that of the control group (AM0) over two consecutive years. The addition of biochar to soil can influence microbe–plant linkages and root colonization patterns, thereby affecting plant root nutrient absorption and promoting plant growth. The potential mechanisms include: (1) The stable carbon in biochar may serve as a microbial energy source, enhancing symbiotic relationships (e.g., with mycorrhizal fungi) and improving plant nutrient uptake efficiency; (2) The porous structure of biochar provides microbial habitats, increasing rhizosphere microbial diversity [3]; (3) Biochar improves soil aeration and water retention, potentially stimulating root development (e.g., increasing root hair density), which subsequently alters the physical space for microbial colonization and modifies exudate composition [5]. Notably, in the second year, the biomass under AM1.5 treatment was 2.59 times higher than that of AM0. Conversely, AM4.5, with the highest application rate, exhibited a 20.6% reduction in biomass compared to AM0 in the second year. These findings are consistent with the conclusions of Liao et al. [28], suggesting that excessive biochar application can diminish its yield-enhancing effects, which exhibit a threshold response [29]. Excessive biochar application elevates the soil C/N ratio, potentially leading to N fixation through heightened adsorption, thereby reducing N availability for plant roots [30]. Thus, biochar as a soil amendment should be applied within a reasonable range to avoid the overprotection of N, which could hinder crop uptake and reduce yields. Based on the results of this study, in the alkalized meadow grassland in the Horqin region, a biochar application rate not exceeding 15 t/hm^2^ (AM1.5) is recommended to achieve optimal yield improvement.

### 4.2. Effects of Biochar Amendment on the Attributes of Alkalized Meadow Soil

The physical properties of alkalized soils are jointly influenced by soil salinity and sodicity, resulting in compacted and swollen soil structures with poor aeration and water permeability. In current study, the application of varying biochar dosages to alkalized meadow soil significantly increased FC and SP, while reducing BD. The EC of the AM1.5 treatment group increased by 50.1% compared to that of AM0, primarily because of the high content of soluble mineral components (e.g., K, Ca, Na) in biochar [7]. However, as the application rate increased, soil EC exhibited a declining trend: the AM3.0 and AM4.5 treatments showed the reductions of 6.7% and 5.6%, respectively, relative to AM1.5. The impact of biochar on soil EC is primarily manifested in the following aspects: (1) After biochar application, its soluble salts are gradually dissolved and released, directly increasing the ion concentration in the soil solution, thereby elevating the electrical conductivity; (2) The porous structure and high specific surface area of biochar enable the adsorption of soluble salt ions (e.g., Na^+^, Cl^−^, Ca^2+^, Mg^2+^) in the soil, reducing their concentration in the soil solution [7]; (3) Oxygen-containing functional groups (e.g., carboxyl and phenolic hydroxyl groups) on biochar surfaces can interact with base ions through complexation or ion exchange, immobilizing them within pores and inhibiting salt migration [5]; (4) Biochar improves soil aggregate structures and enhances porosity and water permeability, thereby accelerating salt leaching to deeper layers or out of the soil profile [31]. Biochar application can exchange H^+^ and Al^3+^ in soils, moderately elevating the soil pH [32]. In this study, soil pH values slightly increased in the biochar-treated groups, though the differences were not statistically significant. Additionally, biochar amendment significantly improved the nutrients in the alkalized meadow soil (Table 2). This enhancement stems from two mechanisms: (1) biochar itself serves as a carbon source and mineral supplement, and (2) its unique porous structure effectively adsorbs soil nutrients, stimulates microbial activity, and accelerates nutrient transformation rates [7].

Soil enzyme can be used as an important indicator for assessing soil quality and biological activity [33]. Enzymes such as protease, urease, polyphenol oxidase, phosphomonoesterase, dehydrogenase, and glucosidase play vital roles in the transformation of soil C, N, and P [34,35,36]. The AM1.5 treatment group exhibited significantly elevated urease, protease, and phosphomonoesterase activities compared to the control group (AM0), which aligns with findings by Xu et al. [37]. This enhancement is primarily attributed to biochar’s ability to optimize soil properties, increase nutrient availability, and improve microbial habitats, with its porous structure providing favorable microsites for microbial activity [7]. However, in the high-dosage AM4.5 treatment group, the enzyme activities slightly declined. This phenomenon may depend on biochar’s strong adsorption capacity: while adsorbing reaction substrates can facilitate enzymatic reactions, the excessive adsorption of enzyme molecules may shield active sites, thereby inhibiting enzymatic processes [38]. Additionally, this study observed that biochar significantly suppressed soil glucosidase activity. Wang et al. [39] reported a marked reduction in soil glucosidase activity after seven consecutive years of biochar application (40–80 t/hm^2^), with the decline positively correlating with the application rate. Similarly, Feng et al. [40] found that biochar suppressed cellulose-degrading enzymes (e.g., β-glucosidase) while enhancing ligninase activity. A plausible mechanism is that, in high C/N ratio soils, biochar alters microbial metabolic pathways, prompting microbes to preferentially utilize aromatic carbon over polysaccharides. This shift reduces the substrate availability for glucosidase-catalyzed reactions, ultimately inhibiting glucosidase activity.

### 4.3. Effects of Biochar Addition on Soil Functional Microbial Abundance

The application of biochar to alkalized meadow soil significantly increased the abundance of microbial *nifH* genes and exhibited a notable linear relationship with the application rate (Figure 1B). Zhang et al. [41] similarly observed increased *nifH* gene abundance following biochar amendment during their investigation into the improvement mechanisms of degraded black soil. The increase in *nifH* gene is attributed to enhanced carbon availability in biochar-amended soil, which promotes the proliferation of N-fixing microorganisms [42]. However, soil ammonia-oxidizing microorganisms showed limited sensitivity to biochar addition, with no significant differences in microbial *amoA* gene abundance observed across treatments (Figure 1A). The findings of Liu et al. [43], which demonstrated significant inhibition of ammonia-oxidizing archaea but minimal impact on ammonia-oxidizing bacteria gene abundance in cinnamon upland with biochar application, are largely consistent with the conclusions of this study. The underlying mechanism may involve biochar’s chemical adsorption of NH_4_^+^, which reduces substrate concentration for nitrification and consequently restricts the growth of ammonia-oxidizing microorganisms [44]. Furthermore, biochar addition significantly enhanced *phoD* gene abundance in the alkalized meadow soil, indicating its potential to stimulate the proliferation of organic P-mineralizing bacteria, promote organic P mineralization, and improve soil P availability. These results are consistent with the studies by Zhang et al. [45] and Yuan et al. [46].

### 4.4. Effects of Biochar Application on Soil Microbial Community Structure in Alkalized Meadow Soil

Biochar can regulate the micro-environment of soil, thereby influencing soil microbial communities [7]. In this study, the bacterial OTUs in the biochar-treated groups AM1.5, AM3.0, and AM4.5 showed increases compared to AM0, consistent with the report of He et al. [5], suggesting that biochar addition facilitates the increase in bacterial diversity. The dominant bacterial phyla (including Proteobacteria, Actinobacteria, Acidobacteria, Cyanobacteria, Bacteroidetes, and Chloroflexi) across all treatments remained unchanged, aligning with previous studies [5,12,47]. Proteobacteria and Actinobacteria dominated in all sampling sites. These phyla exhibit strong salt tolerance and adaptability to extreme environments, explaining their prevalence in arid and semi-arid soils [48]. Although variations in the relative dominance of these phyla were observed among treatments, none reached statistical significance. This phenomenon may be attributed to the intense saline–alkali stress on bacteria in these soils and the recalcitrant carbon in biochar [39]. Sheng and Zhu [4] similarly reported minimal impacts of biochar on the relative abundance of bacterial phyla in alkaline (pH 7.81) podzolic soils.

The application of biochar induced significant changes in the relative dominance of key bacterial genera across treatments. The relative abundances of *Haliangium* [49] and *Luteitalea* [50] (a genus capable of surviving across a wide range of pH and temperature conditions) increased significantly. Conversely, the relative dominance of RB41 [51] (associated with organic matter decomposition) and *Vicinamibacter* [52] (with chitin degradation potential) decreased markedly. This phenomenon indicates that biochar amendment shifted the bacterial community in alkalized meadow soil from taxa specializing in organic matter transformation to those adapted to high salinity and alkalinity. Concurrently, the abundance of *Tychonema* (a cyanobacterial genus) correlated positively with biochar dosage. Cyanobacteria demonstrate remarkable ecological adaptability, with a prolific presence in arid and semi-arid ecosystems where they perform crucial N fixation functions [53]. These phototrophic microorganisms have evolved exceptional metabolic strategies to maintain robust N_2_ fixation capacity even under extreme water-deficient conditions. The substantial enrichment of *Tychonema* aligns with the observed changes in *nifH* gene abundance in this study. Notably, a previous study indicated that cyanobacteria require more available N compared to other N-fixing bacteria [54]. Thus, the reduced biomass of *A. adsurgens* in the AM4.5 treatment may be linked to the rapid proliferation of *Tychonema* CCAP 1459-11B and its preferential utilization of available N. The CCA analysis revealed that, in addition to soil nutrients, EC is also a significant environmental factor influencing the structure of soil bacterial community. Biochar-induced salt accumulation may disrupt soil structure and aggregates [12], thereby promoting the enrichment in salt-tolerant bacterial taxa.

Notably, our investigation revealed no statistically significant alterations in either the taxonomic composition or relative abundance of soil fungal communities at the phylum level. This observation corroborates previous findings documented by Yao et al. [55]. The dominant fungal phyla included Ascomycota, Basidiomycota, Chytridiomycota, Mortierellomycota, Mucoromycota, and Blastocladiomycota, with Ascomycota exhibiting absolute dominance across all treatments. This aligns with the conclusions from fungal community surveys conducted in various grassland zones by other researchers, suggesting relatively consistent soil fungal composition at a global spatial scale [56]. Biochar addition significantly reduced the relative abundances of saprophytic fungi *Stachybotrys* and *Chrysosporium*. Both genera participate in carbon cycling as decomposers: *Stachybotrys* [57] secretes enzymes to convert cellulose and hemicellulose into sugars, while *Chrysosporium* [58] facilitates humification by degrading lignin-derived aromatic molecules. These results indicate that biochar amendment may influence soil carbon cycling through the alterations in fungal community structure. Concurrently, biochar application significantly decreased the relative dominance of *Penicillium*. Studies have identified *Penicillium* as both a key lignocellulose-degrading fungus [59] and a phosphate-solubilizing microorganism [60], capable of converting insoluble phosphates into plant-available forms. The elevated soil AP levels associated with biochar amendment exhibited potential inhibitory effects on *Penicillium* proliferation, while conversely promoting significant enrichment in *Filobasidium* populations in the soil microbial community. Research has demonstrated that *Filobasidium* [61] effectively degrades diverse organic compounds under varying temperatures, pH levels, and salt stress, while also preventing cryptococcal infections. Notably, the relative abundance of *Stagonosporopsis*, a genus associated with plant pathogens, increased markedly in the AM3.0 and AM4.5 treatments, suggesting that a high biochar application rate may elevate risks of soil-borne fungal diseases [62]. Collectively, the recalcitrant carbon in biochar reduced the relative abundance of saprophytic fungi while increasing that of pathogenic fungi in alkalized soils. While this phenomenon is unlikely to cause widespread plant diseases in natural grasslands with high biodiversity, it should be noted when establishing artificial grasslands through tilling, biochar application, and forage sowing. A multi-species mixed sowing approach (e.g., legumes + grasses) is recommended to mitigate risks. The LEfSe analysis revealed that bacterial communities underwent far more taxonomic shifts than fungi under biochar treatment, indicating greater bacterial sensitivity to soil environmental changes. This implies that biochar amendment in alkalized meadow soils may enhance bacterial competitiveness.

This study preliminarily confirmed the improvement effects of biochar on semi-arid soils through pot experiments. However, due to limitations in research duration and experimental conditions, the measurement of soil and microbial indicators was incomplete. Although the experiment spanned two years, only one year of soil and microbial data were collected. Functional soil microorganisms were limited to N-fixing bacteria, ammonia-oxidizing bacteria, and organic P-mineralizing bacteria, while carbon-fixing-, denitrification-, and sulfur-cycling-related microbial communities were not investigated. Soil mineralization processes, soil respiration, and microbial biomass were also not measured. Additionally, different biochar types vary in pH and particle size, yet no comparative experiments were conducted to analyze their differential improvement effects.

This study found that biochar application increased the yield of *A. adsurgens* but reduced yield at higher doses. The underlying mechanisms warrant further investigation, particularly regarding plant root exudates and microbial community responses to biochar treatment. Furthermore, the biochar application rate proposed based on pot experiments should be validated through field trials to determine precise techniques. When selecting biochar types, special attention should be paid to its inherent pH, with a preference for low pH biochar to optimize soil remediation in alkaline grasslands.

## 5. Conclusions

This investigation revealed that biochar amendment in alkalized meadow soils significantly enhanced the biomass production of cultivated *A. adsurgens* while concurrently ameliorating key soil physicochemical parameters. Notably, the study identified a nonlinear dose–response relationship, with excessive biochar application progressively compromising the observed agronomic benefits. Biochar addition significantly reduced soil BD; increased SP, SM, and FC; and simultaneously improved soil nutrients (including SOM, TN, AP, and AK). Additionally, the activities of soil urease, phosphomonoesterase, protease, and polyphenol oxidase significantly increased. Biochar application induced microbial community restructuring in alkalized meadow soils, primarily by modulating the relative dominance of prevalent taxa rather than causing fundamental alterations in predominant phyla or genera. In addition, biochar application significantly increased the quantities of *phoD*- and *nifH*-carrying microbes, suggesting the enhancement in functions of soil N fixation and P transformation; however, the quantity of soil *amoA*-harboring microorganisms was little affected. Key factors influencing bacterial community structure under biochar treatment included EC, TP, TK, BD, and AK, whereas fungal communities were primarily affected by BD, SP, and NH_4_-N. Considering plant biomass increment, soil physicochemical properties, biological activities, the structure shifts of microbial communities, and functional gene abundance, a biochar application rate of 1.5 kg/m^2^ is recommended during the plowing, harrowing, and reseeding stages of forage grass establishment for the restoration of alkalized meadow grasslands.

## Figures and Tables

**Table 1:** Aboveground biomass of *Astragalus adsurgens* according to different treatments (dry weight, kg/m^2^).

Year	AM0	AM1.5	AM3.0	AM4.5	ANOVA in Response to Biochar Application Rate			
					Regression Equation	*R* ^2^	*F*	*p*
1	0.31 ± 0.02a	0.38 ± 0.03a	0.48 ± 0.03b	0.44 ± 0.02b	*y* = 0.064*x* + 0.656	0.714	24.906	<0.001
2	0.34 ± 0.09a	0.88 ± 0.05c	0.55 ± 0.08b	0.27 ± 0.06a	-	0.052	0.548	0.476

Values are means ± SD. AM0: Control; AM1.5, AM3.0, and AM4.5: 1.5, 3.0, and 4.5 kg/m^2^ treatments, respectively. *R*^2^, *F*, and *p* values from regression analysis are presented. Means in row followed by the different letters are significantly different (*p* < 0.05).

**Table 2:** Responses of soil properties to biochar addition.

Index	AM0	AM1.5	AM3.0	AM4.5	ANOVA in Response to Application Rate		
					*R^2^*	*F*	*p*
Soil moisture (%)	6.37 ± 1.05a	9.05 ± 1.14ab	10.77 ± 1.26b	11.22 ± 2.06b	0.664	19.762	<0.001
pH	8.00 ± 0.02a	8.17 ± 0.04a	8.08 ± 0.07a	8.13 ± 0.04a	0.213	2.704	0.131
Organic matter (%)	1.19 ± 0.98a	1.77 ± 0.15ab	2.10 ± 0.57ab	2.31 ± 0.58c	0.579	13.755	0.004
Soil porosity (%)	39.50 ± 1.79a	42.64 ± 2.29b	45.66 ± 1.31c	46.42 ± 0.38c	0.768	33.196	<0.001
Bulk density (g cm^−3^)	1.60 ± 0.05c	1.52 ± 0.06b	1.44 ± 0.35ab	1.42 ± 0.01a	0.768	33.155	<0.001
Field water holding capacity (%)	12.33 ± 2.07a	14.35 ± 1.10b	15.43 ± 2.85bc	16.44 ± 2.27c	0.413	7.030	0.024
Electrical conductivity (μs cm^−1^)	106.1 ± 8.17a	150.2 ± 7.87b	140.2 ± 6.15b	141.8 ± 4.85b	0.372	5.928	0.035
Total N (%)	0.029 ± 0.00a	0.042 ± 0.002b	0.044 ± 0.01b	0.049 ± 0.002b	0.705	23.906	<0.001
Total P (%)	0.02 ± 0.01a	0.03 ± 0.00a	0.04 ± 0.02a	0.04 ± 0.01a	0.283	3.940	0.075
Total K (%)	2.20 ± 0.01a	2.24 ± 0.52a	2.31 ± 0.08a	2.24 ± 0.02a	0.122	1.385	0.266
NH_4_-N (mg kg^−1^)	0.61 ± 0.04a	0.68 ± 0.05ab	0.67 ± 0.03ab	0.75 ± 0.01c	0.640	17.751	0.002
Available P (mg kg^−1^)	15.77 ± 0.18a	26.85 ± 3.18b	38.42 ± 6.03c	42.93 ± 5.63c	0.868	96.818	<0.001
Available K (mg kg^−1^)	147.6 ± 6.91a	221.4 ± 9.90b	205.0 ± 18.76b	216.0 ± 3.96b	0.468	8.811	0.014

AM0: Control; AM1.5, AM3.0, and AM4.5: 1.5, 3.0, and 4.5 kg/m^2^ treatments, respectively. The regression analysis results include the coefficient of determination (*R*^2^), *F*-statistic, and corresponding *p*-values. Statistical significance (*p* < 0.05) between mean values within rows is indicated by distinct letters following the data. All numerical results are expressed as mean ± standard deviation.

**Table 3:** Responses of soil enzymatic activities to biochar addition.

Index	AM0	AM1.5	AM3.0	AM4.5	ANOVA in Response to Application Rate		
					*R^2^*	*F*	*p*
Dehydrogenase (mg TPF kg^−1^ 24 h^−1^)	192.0 ±19.46c	190.4 ± 22.69c	166.6 ± 6.25b	139.7 ± 36.11a	0.494	9.773	0.011
Protease (mg Tyr g^−1^ 2 h^−1^)	75.33 ± 10.41a	107.6 ± 18.24c	88.51 ± 8.70b	92.59 ± 16.98b	0.050	0.528	0.484
Urease (mg 100 g^−1^ 24 h^−1^)	6.30 ± 0.69a	18.66 ± 3.38c	14.37 ± 4.65b	14.82 ± 2.59b	0.209	2.648	0.135
Polyphenol oxidase (µmol g^−1^ 10 min^−1^)	0.686 ± 0.22a	3.31 ± 0.36c	4.6 ± 0.47d	2.70 ± 0.03b	0.338	5.104	0.047
Phosphomonoesterase (mg g^−1^ h^−1^)	76.27 ± 6.43a	118.7 ± 14.01c	97.80 ± 19.71b	85.87 ± 15.46b	0.002	0.020	0.891
Glucosidase (μg g^−1^ h^−1^)	1.17 ± 0.21b	0.74 ± 0.22b	0.54 ± 0.18a	0.54 ± 0.18a	0.722	25.96	<0.001

Values are means ± SD. AM0: Control; AM1.5, AM3.0, and AM4.5: 1.5, 3.0, and 4.5 kg/m^2^ treatments, respectively. The regression analysis results include the coefficient of determination (*R*^2^), *F*-statistic, and corresponding *p*-values. Statistical significance (*p* < 0.05) between mean values within rows is indicated by distinct letters following the data. All numerical results are expressed as mean ± standard deviation.

## Data Availability

The original contributions presented in the study are included in the article. Further inquiries can be directed to the corresponding author.
